# Triple-nerve decompression surgery for the treatment of painful diabetic peripheral neuropathy in lower extremities: A study protocol for a randomized controlled trial

**DOI:** 10.3389/fneur.2022.1067346

**Published:** 2022-12-14

**Authors:** Chenlong Liao, Shuo Li, Xin Nie, Yayuan Tian, Wenchuan Zhang

**Affiliations:** ^1^Department of Neurosurgery, Shanghai Ninth People's Hospital, Affiliated to Shanghai Jiao Tong University School of Medicine, Shanghai, China; ^2^Biostatistics Laboratory of Clinical Research Unit, Shanghai Ninth People's Hospital, Affiliated to Shanghai Jiao Tong University School of Medicine, Shanghai, China

**Keywords:** nerve decompression, diabetic peripheral neuropathy, neuropathic pain, randomized controlled trial, visual analog score

## Abstract

**Objectives:**

Painful diabetic peripheral neuropathy (DPN) is often refractory to conventional medications. Triple-nerve decompression was proposed for painful DPN due to the frequent involvement of multiple nerve entrapments in diabetes. However, the role of decompressive surgery remains controversial. This trial aims to assess the efficacy of triple-nerve decompression for patients with painful DPN suggestive of nerve entrapment using a randomized controlled trial (RCT) design.

**Methods and analysis:**

This trial is a single-center RCT and will be conducted in Shanghai Ninth People's Hospital. Enrolled subjects (*n* = 74) with painful DPN due to nerve compression, which can be detected by nerve conduction studies, will be randomly allocated at a 1:1 ratio into surgical and non-surgical groups. The primary outcome will be measured by 50% responder rates, which is defined as the proportion of subjects with at least 50% reduction of the mean weekly visual analog score (VAS) of pain from baseline after 6 months of treatment. Mean weekly VAS will be additionally evaluated 1 week (W1), 1 month (M1), and 3 months (M3) after treatment to monitor the changes in pain intensity. The secondary outcomes include two-point discrimination (TPD), Toronto clinical scoring system (TCSS), electrophysiological indexes, hospital anxiety and depression scale (HADS), and the medical outcome study short-form 36-item questionnaire (SF-36). A quantitative analgesic questionnaire (QAQ) will be used as a secondary outcome to quantify the analgesic medication weekly. TPD and TCSS will be conducted at W1, M1, M3, and M6 after treatment. Electrophysiological tests, HADS, and SF-36 will be performed at M3 and M6.

**Ethics and dissemination:**

Ethics approval has been obtained from the Ethics Committee of Shanghai Ninth People's Hospital (SH9H-2-21-T323-2). It was registered on the Chinese Clinical Trial Registry website (http://www.chictr.org.cn) on 16 August 2021 with the number ChiCTR2100050049. Written informed consent will be obtained from all participants. The results of this trial will be disseminated *via* peer-reviewed journals, mass media, and presentations at national and international academic conferences.

## Introduction

Diabetic peripheral neuropathy (DPN) is one of the most common complications of diabetes, affecting up to 50% of patients with diabetes (1). The clinical manifestations of DPN vary greatly and the involvement of the lower extremities is more common than that of the upper extremities. As the most distressing symptom of DPN, neuropathic pain adversely affects the quality of life and is the main reason prompting patients to seek medical treatment (2).

The DPN has long been viewed as a progressive and irreversible disorder. In addition to intensive glycemic control and lifestyle modification, the management of painful DPN remains largely symptomatic. However, the efficacy of conventional medication therapy is limited, and the side effects are usually unbearable (3). Faced with this dilemma, surgical interventions, such as nerve decompression and spinal cord stimulation, had been developed as alternative treatment options for painful DPN. Triple-nerve (posterior tibial nerves, common and deep peroneal nerves in bilateral lower extremities) decompression was proposed based on the following observations from either animal or clinical studies: (1) Peripheral nerves in diabetes are susceptible to chronic compression (4, 5); (2) sites of nerve compression in lower extremities have been identified in anatomic studies (6–8); and (3) axons are more prone to be compressed distally when a proximal compression is present along the same axon, and vice versa (“double-crush” theory) (9). Furthermore, at the molecular level, the neuronal response to hyperglycemia through the sorbitol-aldose reductase or polyol pathway should be appreciated to understand the mechanism of nerve compression in the diabetic population. The sorbitol pathway begins with aldose reductase; an enzyme needed to process cellular glucose for energy production. It makes only a small proportion of sorbitol from residually available glucose under physiological conditions. In chronic hyperglycemia, the hexokinase enzyme, which converts the bulk of intracellular glucose to glucose-6-phosphate, becomes fully saturated, and excess glucose is converted to sorbitol by aldose reductase. Due to its characteristic of low plasma membrane permeability, sorbitol acts as an osmotic driver, pulling extracellular fluid into the neuron and causing axonal and nerve trunk swelling (10–12). The swollen nerve in patients with diabetes had been confirmed with the use of diagnostic ultrasound in previous morphological researches (13, 14).

It is well-established that peripheral neuropathy in diabetes is heterogeneous, consisting of a diverse group of syndromes. Entrapment neuropathies are highly prevalent in the diabetic population; nearly, one of three patients has one. Since the treatment might be surgical, entrapment neuropathies should be actively sought in every patient with the presentation of neuropathy (15, 16). Nerve decompression has been advocated for the alleviation of pain, restoration of sensory, and prevention of ulceration in the previous reports (17–21). Furthermore, acute improvement in intraoperative electromyographic recordings following nerve decompression in patients with symptomatic diabetic sensorimotor peripheral neuropathy had also been reported (22, 23). Nevertheless, the role of decompressive surgery in the management of painful DPN remains controversial since the previous studies were mostly retrospective case series, and the level of evidence was deemed inadequate (24, 25). Therefore, a randomized controlled trial (RCT) with high-level evidence was strongly recommended to determine whether patients with painful DPN would benefit from this procedure (26).

According to the literature review regarding nerve decompression for painful DPN, only two RCTs were identified. In the first trial reported by Macare van Maurik et al. from the Netherlands, surgical decompression was performed in a randomly lower limb, and the contralateral one served as a control. Pain improvement in the operated leg was observed in 73.7% of 42 patients 1 year after surgery (27). Similar positive results were also demonstrated in another single-blinded, parallel-group RCT conducted by Best et al. from Canada, in which 22 patients with painful DPN were included and randomized to either surgery (*n* = 12) or observation (*n* = 10) (28). In their study, pain was the primary outcome assessed with two measures, McGill pain visual analog scores and Neuropathy-Specific Quality of Life Scale (NeuroQol) pain item. The former changed significantly over time within the groups, and the NeuroQol pain sensitivity analysis significantly changed from baseline to 12 months, comparing intervention (nerve decompression) to control. In these two RCTs, standard medication treatments including analgesics were allowed to use if needed; however, the medication treatments were not recorded and assessed. To address this limitation, the Quantitative Analgesic Questionnaire (QAQ) will be used in our present trial to quantify the analgesic medication regimen and thus to preclude the confounding effects of analgesics on evaluating the efficacy of nerve decompression for pain relief in DPN (29). Furthermore, the assessment of sensory profiles, psychological status, and quality of life will be conducted in this clinical trial to comprehensively investigate the effect of triple-nerve decompression in the treatment of the patient with painful DPN in the lower extremities. In this study, triple-nerve decompression will be compared with conventional medication for clinical effectiveness in the treatment of painful DPN. We believe that the completion of this clinical trial would add solid evidence to the literature.

## Methods

### Study design and setting

**Figure 1:**
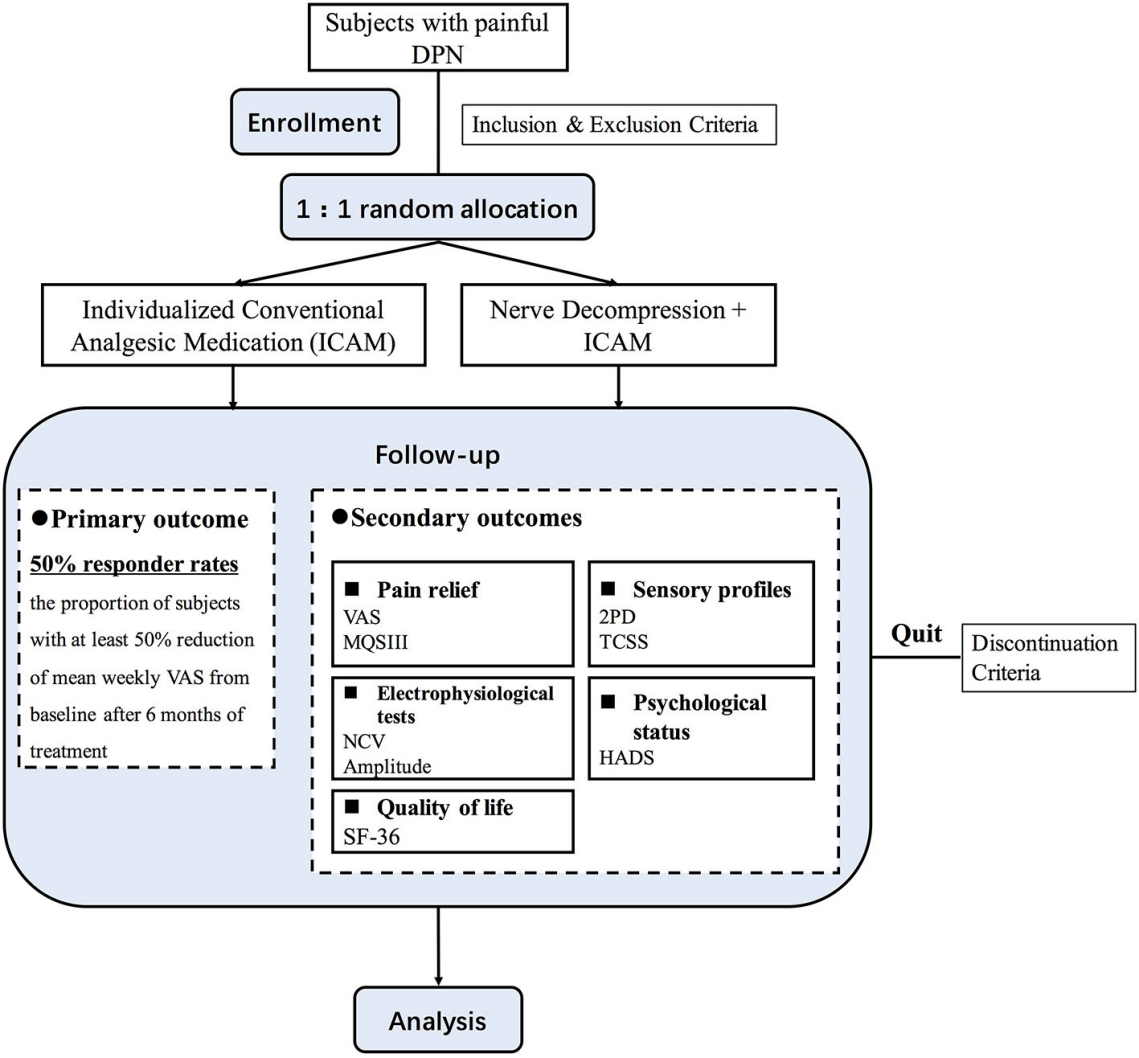
A study flow diagram.

This trial was designed in accordance with the Standard Protocol Items: Recommendations for Interventional Trials (SPIRIT) (30). This study is a single-center RCT and will be conducted in Shanghai Ninth People's Hospital, Shanghai Jiao Tong University School of Medicine. Participants will be recruited from the Department of Neurosurgery. Before enrollment, a diagnosis of painful DPN will be made based on the results of medical history, physical examination, and electrophysiological examination of peripheral nerves. After signing an informed consent, subjects will undergo a complete evaluation, including medical history taking, physical examination, blood tests, and electrophysiological examination. Details of the inclusion and exclusion criteria were provided in Table 1. As illustrated in Figure 1, subjects meeting the inclusion and exclusion criteria will be randomly allocated at a 1:1 ratio into surgical and non-surgical groups. The assessments and follow-up plans, in this study, are summarized in Table 2. Previous medication treatment for neuropathy will be required to be stopped for at least 1 week before being formally enrolled in this trial.

**Table 1:** The criteria for inclusion, exclusion, and discontinuation.

**Inclusion criteria**
1. Diagnoses
Diagnosed with confirmed diabetes according to the 1999 WHO diagnostic criteria for type 2 diabetes
2. Age
40–70 years old
3. Neuropathy
Michigan Neuropathy Screening Instrument (MNSI) clinical examination score > 2
4. Blood glucose control
Fasting blood glucose < 8 mmol/L and glycosylated hemoglobin (HbA1c) < 8%
5. Neuropathic pain
i. Neuropathic pain is distributed in the sensory areas innervated by the common peroneal nerve, posterior tibial nerve, and deep peroneal nerve
ii. Pain symptoms last for more than 6 months
iii. VAS of neuropathic pain is higher than 4
6. Nerve electrophysiology
Decreased nerve conduction velocity presented in more than one nerve (common peroneal nerve, posterior tibial nerve, and deep peroneal nerve) of the lower extremities (≥2 standard deviations)
**Exclusion criteria**
1. Existence of medical history that would cause neuropathy
i. Comorbidities such as pernicious anemia, hypothyroidism, vascular disease of the lower extremities (disappeared or weakened of the pulse of dorsalis pedis artery, varicose veins, etc.),
ii. Traumatic or surgical history of the lower extremities
iii. Medication history such as vincristine, phenytoin, etc.
iv. History of heavy metal poisoning, etc.
2. History of alcohol or drug addiction or abuse within 1 year at the time of enrollment
3. Lumbar spinal stenosis or active radiculopathy from lumbar disc herniation
4. Edema of lower extremities caused by various reasons
5. Existence of severe hemorrhagic diseases or bleeding tendency.
6. Intolerant to surgery or analgesics due to dysfunction of heart, lung, liver, kidney, and other vital organs
7. Patients with mental disorders and other factors that affect compliance
8. Rheumatism and rheumatoid diseases
9. Patients who are participating in other clinical trials
10. Previous foot ulceration or amputation
**Discontinuation criteria**
1. Occurrence of severe complications, side effects, comorbidities or other special physiological changes, such as pregnant, that prevent the subjects from continuing to participate
2. Lack of compliance to the protocol by the subjects that render the data collection unreliable
3. Loss to follow-up

**Table 2:** Study protocol timeline per subject.

**Timeline**	**Screen phase^†^**	**Baseline**	**Follow-up phase**			
**General characteristics**	**1st visit**	**2nd visit**	**3rd visit/W1**	**4th visit/M1** ^‡^	**5th visit/M3** ^‡^	**6th visit/M6** ^‡^
Informed consent	•					
Demographic data	•					
Clinical diagnoses	•					
Medical records	•					
Vital signs	•		•	•	•	•
Physical examination	•		•	•	•	•
**Laboratory tests**						
Fasting blood glucose	•			•	•	•
HbA1c	•			•	•	•
Routine blood test	•			•	•	•
Routine coagulation test	•			•	•	•
Blood tests for hepatic and renal function	•			•	•	•
**Criteria**						
Inclusion criteria	•					
exclusion criteria	•					
**Treatment**						
Surgery		•				
Medication		•				
Complication		•	•	•	•	•
**Outcomes**		•				
VAS	•	•	•	•	•	•
QAQ^§^		•	•	•	•	•
Two-point discrimination	•	•	•	•	•	•
TCSS		•	•	•	•	•
Electrophysiological test	•				•	•
HADS		•			•	•
SF-36		•			•	•
Adverse events		•	•	•	•	•

† Subjects will be enrolled within 2 weeks after the screening phase. The results of blood tests and examinations within 14 days before signing the informed consent are acceptable in the screening phase.

‡ Both groups will be followed up at the same frequency after treatment. The time horizon of the follow-up is 14 days, that is, the patients are followed up within 7 days before and after the follow-up time point.

§ QAQ will be used to quantify the analgesic medication regimen weekly.

### Study goals and objectives

The purpose of this study is to examine the role of decompressive surgery in selected patients with painful DPN suggestive of nerve entrapment. The primary objective of this clinical trial is to evaluate the efficacy of triple-nerve decompression for relieving neuropathic pain of DPN in the lower extremities. In addition, this research will investigate whether restoration of co-existed dysesthesia, improvement of nerve electrophysiology, psychological status, and quality of life could be better achieved by nerve decompression compared with conventional medication. The analgesic medication regimen will be quantified and analyzed with QAQ, the results of which will serve as an adjunctive evaluating indicator of the efficacy of nerve decompression (29).

#### Primary outcome

The primary outcome will be measured by 50% responder rates, which is defined as the proportion of subjects with at least a 50% reduction of the mean weekly visual analog score (VAS) of pain from baseline after 6 months of treatment in this trial. Mean weekly VAS will be calculated from the average VAS within the last 7 days. The subjects will be instructed to describe the intensity of their pain using an 11-point scale ranging from 0 to 10, with 0 as “no perceptible pain” and 10 as “unbearable pain.” When the pain is distributed in bilateral lower limbs, VASs of both lower limbs will be averaged together to generate a mean pain score. Mean weekly VAS will be, in addition, evaluated 1 week (W1), 1 month (M1), and 3 months (M3) after treatment to track changes in pain intensity.

#### Secondary outcomes

##### QAQ

The QAQ is an instrument designed for the comprehensive quantification of patient-reported chronic pain medication regimens, including the calculation of percent adherence and generation of quantitative scores to estimate and track changes in medication use over time (29). The application of QAQ will be helpful for point assessment of individual subjects and tracking pain levels throughout the trial period.

##### Two-point discrimination

**Figure 2:**
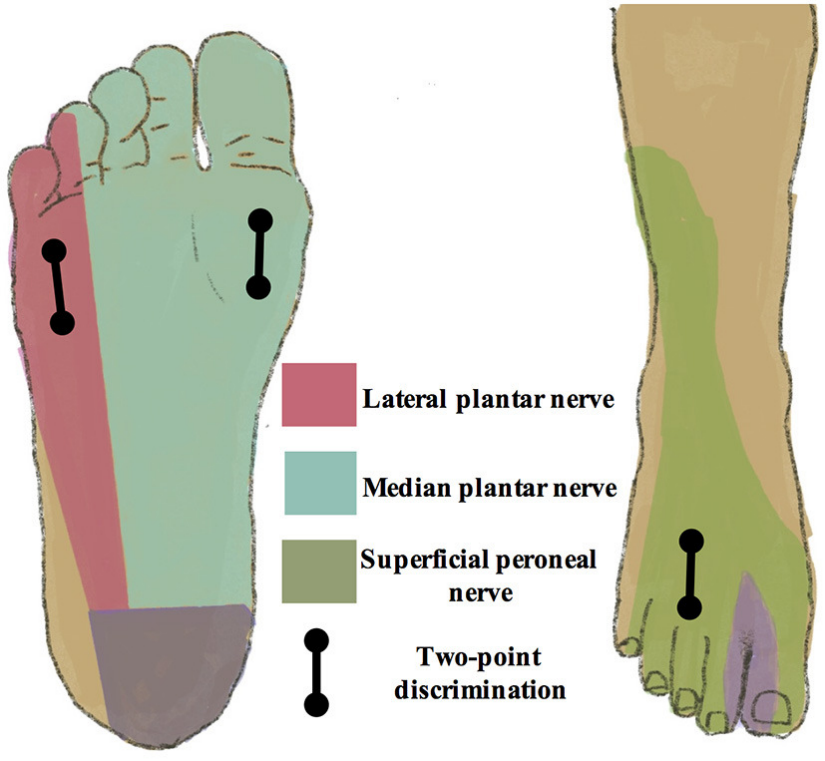
Sensory territories of peripheral nerves in the lower extremities. The two-point discrimination test is performed in the sensory territories of the superficial peroneal nerve, medial, and lateral plantar nerves, including the anterior dorsum of the foot, and medial and lateral anterior plantar area.

The TPD test is a quantitative assessment of tactile acuity, discriminating between the sensation of two points touching the skin surface and that of only one point. It has been reported to be a simple and cost-effective clinical examination to evaluate the onset and progression of diabetic neuropathy (31). In this trial, the TPD test is performed in three locations, including the anterior dorsum of the foot and the medial and lateral anterior plantar area. These three areas are correspondingly located in the sensory territories of the superficial peroneal nerve, and medial and lateral plantar nerves (Figure 2). The TPD test is performed according to the method described by Moberg (32).

##### Toronto clinical scoring system

The TCSS was initially developed as a clinical stratification method in a study of simple screening tests for DPN (33). Later on, the TCSS was validated as an instrument to reflect the presence and severity of DPN (34). There are six symptom scores (the presence or absence of foot pain, numbness, tingling, weakness, imbalance, and similar upper-limb symptoms), eight scores of deep tendon reflexes (bilateral knee and ankle reflexes, each graded as absent, reduced, and normal), and five physical examination scores performed at the first toe (the presence or absence of pinprick, temperature, light touch, vibration, and position perception). Grading of TCSS is as follows:≤ 5 indicates no neuropathy, 6–8 indicates mild neuropathy, 9–11 indicates moderate neuropathy, and ≥12 indicates severe neuropathy.

##### Electrophysiological tests

As described in our previous study (17), the electrophysiological tests are conducted by two skilled technicians with the employment of a Denmark Medtronic electromyogram in an indoor environment with a room temperature of 25°C. In the supine position, the subjects are instructed to relax their lower limbs, and their skin temperature is kept higher than 32°C. Bilateral motor nerve conduction velocity (NCV) and amplitude of both posterior tibial nerve and common peroneal nerve are conducted. Bilateral sensory NCV and amplitude of superficial peroneal nerve are performed.

##### Hospital anxiety and depression scale

Subjects are instructed to complete a questionnaire on HADS, which is a self-reported questionnaire to assess the psychological status of both anxiety and depression. HADS is developed for the non-psychiatric clinical setting and includes 14 items: half items for anxiety (HADS-A) and half for depression (HADS-D). Ranging from 0 to 21, the total score indicates the degree of either anxiety or depression: 0–7 indicate asymptomatic cases, 8–10 indicate doubtful cases, and 11–21 indicate definite cases (35).

##### The medical outcome study short-form 36-item questionnaire

SF-36 is widely used to assess the quality of life across a wide spectrum of diseases, including painful DPN (36). It is a self-reported questionnaire that includes eight subscales: physical functioning (PF), role-physical (RP), bodily pain (BP), general health (GH), vitality (VT), social functioning (SF), role-emotional (RE), and mental health (MH). These eight subscales construct physical and mental health summary measures (37).

Along with the mean weekly VAS, TPD and TCSS will be conducted at W1, M1, M3, and M6 after treatment. Electrophysiological tests, HADS, and SF-36 will be performed at M3 and M6, and QAQ will be used to quantify the analgesic medication regimen weekly (Table 2).

### Randomization

Randomization will be performed with the use of a blocked randomization protocol by an independent statistician (NX) to balance the number of subjects in each treatment group. The randomization results are kept in sealed opaque envelopes, which are sequentially numbered and locked in a secured box by a nurse specially assigned. After completion of individual subject enrollment, a corresponding envelope will be opened by an enrollment nurse in front of the subject, and the allocation status will be recorded. Owing to the nature of the treatments, blinding the subjects and surgeons for the allocation status is not feasible. Enrollment nurses and surgeons will not be involved in outcomes assessment, and all investigators involved in data analysis will be blinded to the allocation status.

### Interventional procedure

**Figure 3:**
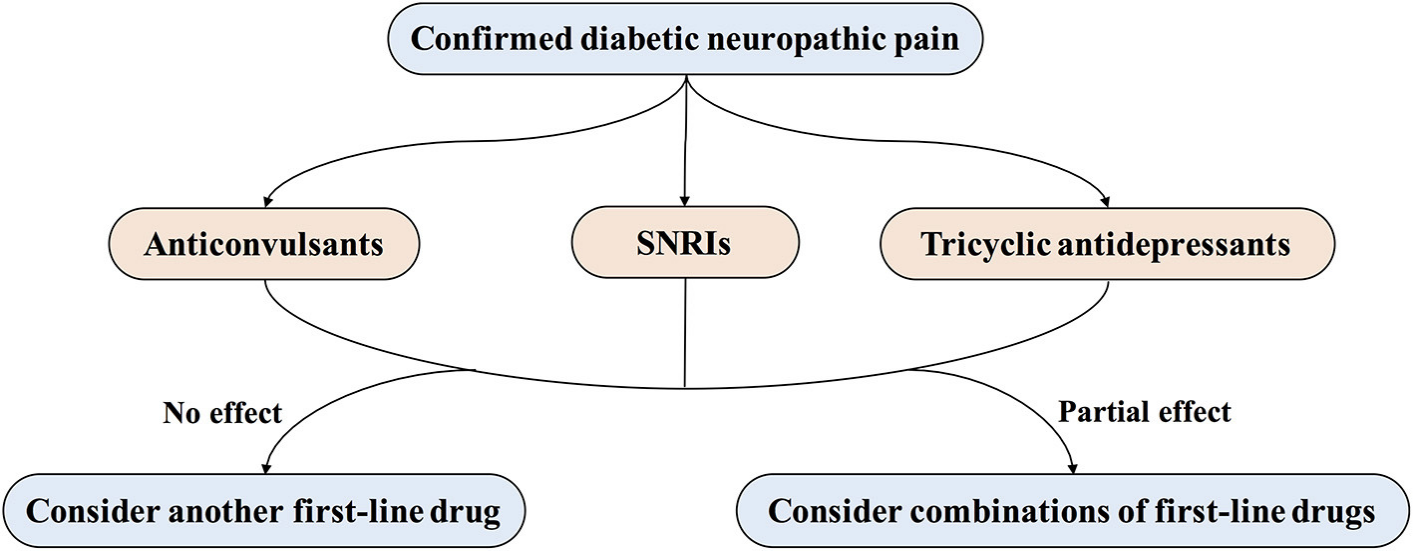
Treatment of painful diabetic peripheral neuropathy. First-line treatments for painful DPN include several drug classes, such as anticonvulsants (pregabalin or gabapentin), serotonin and noradrenaline reuptake inhibitors (SNRIs; duloxetine), and tricyclic antidepressants (amitriptyline).

Subjects in the non-surgical group are treated with conventional medication for diabetes and pain symptoms. The analgesic medication will be prescribed according to the consensus from multiple guidelines and systematic reviews (38–40). First-line medications for diabetic neuropathic pain include calcium channel a2δ ligands (Pregabalin or Gabapentin), serotonin and noradrenaline reuptake inhibitions (SNRIs) (Duloxetine), and tricyclic antidepressants (TCAs) (amitriptyline). For individualized treatment, comorbidities, potential drug interactions, and severity or frequency of side effects will be taken into consideration when choosing the same class of analgesics (Figure 3) (11, 41). The common doses and side effects of each drug are listed in Table 3 (42). The analgesic medication regime in each subject will be assessed with the use of QAQ (29).

**Table 3:** Pharmacotherapies for painful DPN.

**Classes**	**Drugs**	**Dose ranges**	**Side effects**
Anticonvulsants	Pregabalin	Up to 100 mg TID, escalate to 100 mg TID within 1 week of initiation, escalate to 300 mg BID after 2–4 weeks when insufficient pain controlled	Somnolence, blurred vision, dry mouth, edema, weight gain
	Gabapentin	1,800–3,600 mg/day, starting from 300 mg QD, increase to 300 mg BID and TID, then escalate at TID	Dizziness, fall, somnolence, edema, gait disturbance
SNRIs	Duloxetine	60 mg QD	Nausea, somnolence, decreased appetite, fatigue, constipation
TCA	Amitriptyline	10–150 mg/day, increase by 10–25 mg/day every 3–7 days based on tolerability	Orthostatic hypertension, dry mouth, urinary retention, constipation

Subjects in the surgical group will undergo triple-nerve decompression procedure within 2 weeks after randomization. Throughout the study period after surgery, subjects are allowed to use analgesics if they still suffer from diabetic neuropathic pain, which will be carefully assessed and differentiated from postoperative short-term incision pain. As described in our previous study (17), Dellon's triple-nerve decompression of posterior tibial nerves and common and deep peroneal nerves in bilateral lower extremities will be performed under general anesthesia by the same senior surgeon (Zhang WC) (43). The procedures are briefly described as follows.

A curved incision will be made along the medial malleolus, followed by opening the total length of the flexor retinaculum to expose the underlying tibial nerve and vessels. The tibial nerve will be followed distally to reach its division into lateral, medial, and calcaneal branches. With retraction of the abductor hallucis muscle, the deep fascia and the septum between medial and lateral plantar nerves are cut, and the underlying nerves are thus released. A second incision is made longitudinally over the dorsum of the foot. The tendon of the extensor hallucis brevis muscle is exposed and excised to decompress the underlying deep peroneal nerve. A third incision is made obliquely across the fibular neck and exposes the deep fascia, which is lifted to identify the common peroneal nerve. With the peroneus longus muscle retracted anteriorly, the common peroneal nerve is decompressed by dividing the deep fascia and releasing a frequent fascial band beneath the muscle. Postoperatively, patients are instructed to flex and extend the ankles and knees for 3 weeks. Unburdened mobilization will not be permitted until 3 days later, and the use of a walker aid is encouraged.

### Sample size

According to previous reports, the 50% responder rate of nerve decompression plus conventional analgesics medication is about 85% (20, 44), while conventional analgesics medication is about 50% (42). Given a type I error probability of 5% and a 10% loss to follow-up, a sample size of 37 in each group (ratio = 1:1) is estimated to provide 90% power to detect the statistical difference between surgical and non-surgical groups.

### Follow-up

Subjects in the surgical group will be discharged about 1 week after surgery, and the stitches will be removed about 2 weeks later. As demonstrated in Table 2, the follow-up evaluations will be conducted in the outpatient clinic at four time points: W1, M1, M3, and M6. Electrophysiological tests, questionnaire of HADS, and SF-36 will not be performed at W1 since the time interval is too short for these evaluations. QAQ will be assessed *via* telephone or internet weekly. The time horizon of each visit is set to be 14 days (within 7 days before and after the follow-up time point). Subjects are instructed to contact the investigators promptly in case any adverse events (AEs) occur between the visits.

### Data management and statistical analysis plan

Primary data collection from the source document (medical records) review will be performed with the employment of an electronic data capturing (EDC) system on the electronic case report form (e-CRF). The e-CRF will be completed by the principal investigator (CL) or his designee on a continuous basis from the enrollment to the final follow-up visit. Only the principal investigator or his designee will be authorized to enter data *via* the e-CRF with a unique username and password. Each login to the e-CRF will be recorded so that data operation can be tracked and verified. To ensure the consistency and accuracy of data, the automatic logic checks will be conducted in EDC before manual verification by the data administrator.

Continuous variables with normal distributions will be expressed as means and standard deviations, while median and interquartile ranges will be used for those with skewed distributions. Counts and proportions are used for categorical variables. Categorical data, including gender and responder rates, will be analyzed by Pearson's *X*^2^-tests (or Fisher's exact test, if appropriate). The analysis of continuous data with normal distributions will be conducted using the independent two-sided *t*-test, and continuous data with skewed distributions will be analyzed by the Wilcoxon test. To compare the difference in the VAS within and between the groups over time, a repeated-measurement analysis of variance (MANOVA) is performed. The same method is applied to analyze secondary outcomes, including two-point discrimination, electrophysiological indexes, and the scores of QAQ, TCSS, HADS, and SF-36. Multiple linear regression is employed to investigate the association of both primary and secondary outcomes with baseline characteristics of patients, such as a course of diabetes, DPN, and pain. A two-sided *P* < 0.05 is considered statistically significant. Analyses will be made on the base of intention-to-treat (ITT), and per protocol, the analysis will be made for secondary outcomes when no statistical significance is reached by ITT. Data analysis is performed with the employment of statistical software (IBM SPSS Statistics, version 25.0, IBM Corp).

### Safety considerations

For each subject enrolled, all the AEs will be recorded in detail from the beginning to the last visit. According to our and others' prior reports, the main complication of nerve decompression is the issue of incision healing, which can be well-managed by active dressing change (17, 27, 28, 45). The common AEs of analgesics include dizziness, drowsiness, ataxia, and so forth. The symptoms would be effectively reversed by changing or withdrawing analgesic medication (42). The data associated with the clinical safety of the study will be monitored quarterly by the Data Safety Monitoring Board (DSMB). Unanticipated serious AEs will also be reviewed by the DSMB on an urgent basis if needed. DSMB members are independent of this trial, consisting of physicians from different institutes in China. The reports of AEs are regularly submitted to the Institutional Review Board, and the decision will be made on whether the trial can be continued.

### Quality assurance

Investigators in this study followed accredited good clinical practice (GCP) training, and the study was conducted in accordance with GCP regulations. Any deviation from the original protocol must be reported, and the reason must be documented. To ensure the consistency of study procedures, all investigators will participate in a training meeting before the start of the study. After the first patient is enrolled, the files and data will be checked quarterly by the quality control group of institutional Clinical Research Unit (CRU). At the end of the trial, all the files and data will be sealed and archived at a secure location. The Chinese version of this TRENDS study protocol had been anonymously reviewed and approved to be funded by an invited expert panel from the major hospitals in Shanghai, and this project was thus funded by the Medical Innovation Research Project of the Science and Technology Innovation Plan of Shanghai Science and Technology Commission (21Y11906300). The fund will be inspected at the end of each year by the institutional CRU, which is independent of the research team.

### Project management

The neurosurgeons, endocrinologists, and neurologists will recruit the subjects. The principal investigator will check for eligibility and introduce the study process, including the detailed schedule, potential benefits, and risks. Neurologists and endocrinologists will perform the physical examination. Neurosurgeons will gather consent signatures and perform surgical procedures and data collection in the follow-up period. The neurosurgeons, endocrinologists, and neurologists will be available for the consults of these respective specialties throughout the trial period.

## Discussion

According to a practice advisory by the American Academy of Neurology, the efficacy of nerve decompression for painful DPN remained unproven due to the lack of sufficient evidence (24). Numerous mainstream academic groups, including American Diabetes Association, are strongly advocating for clinical trials with high-level evidence to determine whether surgical decompression is beneficial (25, 26, 46). The present trial is conducted based on our previous clinical and translational studies, in which we investigated the factors associated with a favorable surgical outcome and explored the pathological mechanisms underlying painful DPN (17, 35, 47, 48). According to our previous clinical practice, diabetic patients with higher VAS are more likely to seek surgical consultants than those with lower VAS. Therefore, the inclusion criterion for VAS of neuropathic pain is set to be higher than 4 in the present study to facilitate subject recruitment. However, it should be noted that patients with lower VAS might also benefit from nerve decompression, and this set of patients might also suffer from numbness and be at more risk of amputation.

DPN is a collection of diverse clinical disorders, and the causes of neuropathy were likely multifactorial, such as compressive and metabolic impairments (49, 50). It should be noted that decompressive surgery targets the superimposed nerve compression and may not ameliorate the symptoms resulting from other pathological factors than compression. Therefore, the beneficial effects of surgical decompression of nerves in the lower extremities are reserved for a selected group of patients, and identification of nerve compression is of vital importance to improve the efficacy of nerve decompression for the treatment of painful DPN. In the present study, electrophysiological tests will be employed as a primary screening instrument to confirm nerve entrapment, which is mainly indicated by decreased NCV. We expect to obtain sufficient evidence from this trial to justify the role of nerve decompression in the treatment of painful DPN as we claim previously that “when there is compression, there should be decompression” (51).

## Ethics statement

The study protocol and informed-consent form were reviewed and approved by the Ethics Committee of Shanghai Ninth People's Hospital, Shanghai Jiao Tong University School of Medicine Ethics Committee (SH9H-2-21-T323-2). This trial will be conducted according to the principles of the Declaration of Helsinki. Information regarding this trial will be provided verbally and in writing to the participants, who will be given sufficient time to consult the investigator and consider their participation. Participants' autonomy in making informed consent will not be compromised. The informed-consent form should be the signed and dated by the participants as well as the investigators conducting the informed-consent process. A copy of the signed and dated informed-consent form will provide to the participant. The results of this trial will be disseminated *via* peer-reviewed journals, mass media, and presentations at national and international academic conferences.

## Author contributions

CL obtained funding. CL and WZ designed the study. XN developed the statistician analysis plan. SL and CL were involved in designing the case report form and revision of the manuscript. XN, YT, and WZ reviewed the manuscript and provided the critical appraisal. CL and WZ drafted the manuscript. All authors read and approved the final manuscript version.
